# CoHet4Rec: A recommendation for collaborative heterogeneous information networks

**DOI:** 10.1371/journal.pone.0313491

**Published:** 2025-04-09

**Authors:** Yao Chen, Yuling Chen, Zhi Ouyang, Haiwei Sang, Hui Dou, Yangwen Zhang

**Affiliations:** 1 State Key Laboratory of Public Big Data and College of Computer Science and Technology, Guizhou University, Guiyang, China; 2 School of Mathematics and Big Data, Guizhou Education University, Guiyang, China; China University of Petroleum East China, CHINA

## Abstract

Recommender Systems (RS) aim to predict users’ latent interests in items by learning embeddings from user-item graphs. Graph Neural Networks (GNNs) have significantly advanced RS by enabling the embedding of graph-structured data. However, relying solely on user-item interactions has limitations, such as the cold-start problem. Social recommendation has gained attention for its potential to improve outcomes by incorporating social information among users. Yet, existing social-aware models need further exploration of interaction semantics and other collaborative relationships beyond social connections. This paper addresses these limitations by proposing CoHet4Rec, a recommendation model leveraging GNNs and a Collaborative Heterogeneous Information Network (CHIN) with latent collaborative heterogeneous relation factors. CoHet4Rec captures diverse connections between users and items through factorized representations, and has the flexibility to easily incorporate more knowledge beyond social networks to alleviate data sparsity and cold-start problem. Extensive experiments on three benchmark datasets demonstrate the superiority of CoHet4Rec over 15 state-of-the-art (SOTA) recommendation techniques. The highest average improvement is 31.88% for HR@5 and 38.39% for NDCG@5.

## Introduction

Recommender Systems (RS), in recent years, have become essential in online applications, tackling information overload by learning personalized user preferences [1,2]. They achieve this by leveraging user-item interaction histories [3]. However, RS that depend exclusively on these histories encounter challenges like sparse interaction data (i.e. most users have only acted on a few items, which can affect the accuracy of the RS) and the cold-start problem (i.e. it difficult to provide effective recommendations for new users or items without sufficient user behavior data). Recommending cold items in RS is a long-standing challenge, as hot items are recommended based on user behavior, while cold items are recommended based on content characteristics [4].

Collaborative Filtering (CF) is a classic machine learning technique in RS (e.g., [5–7]). While traditional CF has been successful, it often struggles with the cold-start problem and sparse interaction data, and it is limited in handling graph-structured data. With the advancement of deep learning, research has shifted toward Graph Neural Networks (GNNs), which excel at capturing complex relationships in graph-structured data and provide a robust framework for learning node embeddings. This has led to the development of GNN-based recommendation models [3,8]. These methods effectively model user-item interaction graphs using graph neural encoders, enhancing user representation learning. However, sparse data and cold-start problem still pose challenges to the performance of prediction models [9].

**Fig 1:**
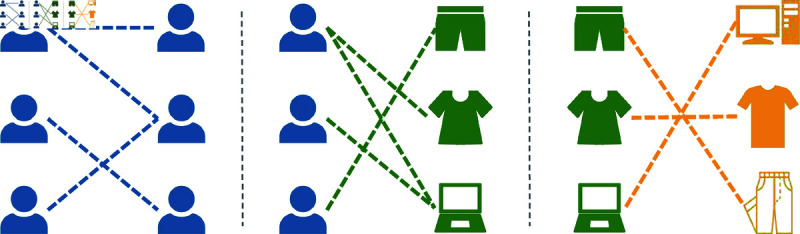
Social network, user-item interaction and item-category relationship.

Previous research [10] has shown that social networks, such as trust links on shopping websites [11], significantly influence users’ online behavior. Combining social links with user-item interactions (Fig 1) enhances recommendation performance [12,13], known as the social recommendation problem. This approach relies on Heterogeneous Information Networks (HINs), which integrate various entities and relationships, strengthening connections between users and items, as well as explore potential collaborative correlations between various entities [14–16]. Researchers hypothesize that users with shared social connections exhibit similar interests. By integrating social information, both social connections and interaction data can be captured simultaneously, improving recommendation accuracy and diversity and mitigating sparse data and cold-start problem. This hypothesis encourages exploring more heterogeneous collaborative relationships beyond the user domain to enhance recommendation accuracy. If validated, it could provide new perspectives for addressing sparse data and cold-start problem in RS, especially when considering the potential performance degradation with privacy-preserving technologies like federated learning and sensitive data collection techniques [17–21].

In real-world recommendation scenarios, significant dependencies exist not only among users but also among items. Users’ preferences for items can be inferred through their social relationships and fine-grained relational knowledge of items, such as item-category relationships (Fig 1) or temporal knowledge (like ODETKE [22]). For instance, user interactions like purchasing, liking, or sharing may be influenced by item features such as brand, color, or director [23,24]. The characteristics of items are composed of many factors such as brand, type, color, and how the item interacts with other behaviors (such as sharing or purchasing between users). In short, there is often a potential connection and mutual influence between the characteristics of users and items. Thus, exploring the rich semantic relationships between users and items is crucial for understanding users’ complex interests [25,26].

Incorporating relational heterogeneity and cross-node dependencies into social recommendation and GNN models is challenging due to the diversity of relationships. One solution is dependency-based heterogeneous graph learning methods [15,27], which integrate structural connections between users and items into graph models. However, these often rely on manually designed meta-paths, requiring domain-specific knowledge and lacking adaptability to different scenarios. Additionally, existing methods typically adjust transformation weights in the feature representation process of node or edge types, limiting their ability to capture the complete heterogeneous relationship context from both user and item perspectives. Most current social recommendation solutions either do not explore or only superficially explore the rich collaborative semantics among items (e.g., ConsistRec [28]). Moreover, with the continuous development of algorithms, data and computing power, the increase in size and complexity of these models has led to an increase in training costs [29].

To address these challenges, we propose a novel recommendation model, CoHet4Rec, based on Collaborative Heterogeneous Information Networks (CHIN). This model integrates various information sources and learns latent features without relying on specific nodes and edges. First, we incorporate user-user social relationships and item-category relationships into RS to extend user-item interactions and construct semantically richer collaborative heterogeneous networks. Then we use GNNs to learn node embeddings. To handle the heterogeneity and collaboration of relationships in the data, we incorporate external collaborative heterogeneous relation units and differentiable embedding propagation operators into the GNN architecture. We introduce an adaptive first-order collaborative heterogeneous neighbor attention mechanism, which combines multiple collaborative semantics from both user and item domains, enhancing user-item interactions. By assigning different attention weights to heterogeneous neighbors, we capture their varying importance. This model does not require manually designed meta-paths, offers high scalability, and can accommodate personalized information dissemination needs by either designing meta-paths or considering broader contextual collaborative semantics.

**In summary, the key contributions of our work are:**

- We provided a new insight into alleviating sparse data and cold-start problem by enhancing user-item interaction and adopting extended heterogeneous relational contexts in both user and item domains.
- We proposed a GNN-based and CHIN-based recommendation framework that no longer relies on manually designed meta-paths to coordinate the context of different information sources.
- Extensive experiments conducted on three real-world datasets have demonstrated that CoHet4Rec outperforms existing methods. For instance, HR@5 and NDCG@5 are increased by 16.00% and 18.18% on average on Ciao dataset.

## Related work

### Social RS

Early approaches in social RS focused on CF techniques and integrating social network information to address the cold-start problem and enhance recommendation diversity. For instance, SoRec [5] decomposed the user rating matrix and social link matrix using coefficients, while HGMF [6] introduced hierarchical group matrix factorization to learn user group features in social networks for recommendations. However, traditional machine learning methods do not perform outstandingly on graph structured data.

Recent advancements in deep learning have propelled social RS, with GNN playing a key role in capturing complex dependencies in social networks. GraphRec [8] and SAMN [30] employed Graph Attention Networks to learn user and item features effectively, while MHCN [31] utilized self-supervised learning for data augmentation. SENGR [32] introduced an emotion-enhanced neural graph recommender that integrates text comments and bipartite graphs, and GNNRec [33] is a conversation-based social recommendation model that simulates users’ interests using their conversation and social data. While these studies highlight the importance of incorporating social information into RS, they often overlook the collaborative aspects of heterogeneous relationships on the item side, limiting their ability to fully exploit diverse user preferences for accurate recommendations. To fill this gap, this work aims to explore a new social RS that explores potential collaborative heterogeneous semantics under GNN architecture.

### HIN-based RS

Heterogeneous graph-based RS is an emerging paradigm that harnesses diverse, multi-relational data across various domains [34,35]. For example, DisenHAN [36] focuses on learning different user/item aspects in a HIN by utilizing meta relationships to decompose high-order connectivity between nodes. DGNN [37] disentangles heterogeneous relationships by propagating information separately and aggregating edges within the same relationship during node aggregation. MML [16] maps users and items to a unified latent vector space, modeling not only the observed relationships between users and items (user-user, item-item, user-item), but also the latent relationships. However, there is still a gap in their exploration of potential collaboration between various relationships.

Most works in this field have used meta-path-based techniques to explore intricate relationships within heterogeneous networks. For instance, a dual attention mechanism was been proposed in HAN [27] to effectively learn the significance of different meta-paths. SMIN [38] designed a meta-path guided heterogeneous GNN that incorporates the relationship structure between social and knowledge awareness into user preference representation. MAHGE [39] proposed a meta-path aggregation method to model time as a relationship on the graph edge, capturing complex dependencies between users and time. MNRec [34] derives user and item embeddings through meta-paths based on domain knowledge and a commuting matrix-based approach for heterogeneous network embedding. Additionally, MAERec [35] employs automatic meta-path extraction from HIN and a hierarchical attention network to learn explicit representations of context-based meta-paths. While meta-path-based methods mentioned above are effective for recommendation in HIN, they often rely on domain experts to create precise meta-paths or complex methods to generate them.

## Preliminary

In this paper, we have three collections: users, items, and categories, represented as $U=\{u_{1},u_{2},...,u_{N}\}$, $V=\{v_{1},v_{2},...,v_{M}\}$, and $C=\{c_{1},c_{2},...,c_{K}\}$, respectively, where *N*, *M*, and *K* are the numbers of users, items, and categories. Social relationships are represented by a social matrix $S\in {\left\{0,1\right\}}^{N\times N}$. Interaction behavior between users and items is captured by an interaction matrix $X\in {\left\{0,1\right\}}^{N\times M}$. Item-category relationships are depicted by a relation matrix $T\in {\left\{0,1\right\}}^{M\times K}$. In matrix *X*, if there is an interaction (e.g., rating) between user *n* and an item *m*, it is denoted as $x_{nm}=1$; otherwise, $x_{nm}=0$. In matrix *S*, a social link between users *n* and $n^{\prime }$ is denoted as $s_{nn^{\prime }}=1$; otherwise, $s_{nn^{\prime }}=0$. It is important to note that a user can have social connections with multiple other users. In matrix *T*, if an item *m* belongs to category *k*, it is denoted as $t_{mk}=1$; otherwise, $t_{mk}=0$. An item can belong to multiple categories.

CoHet4Rec aims to train a recommendation model using *X*, *S*, and *T* to accurately predict user preferences for unseen items.

## Method

**Fig 2:**
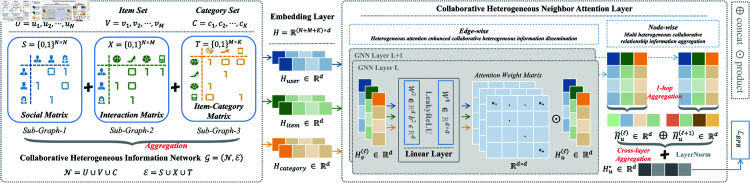
Overall framework of CoHet4Rec. Expanding on the conventional user-item interaction network, we incorporate the social network and item-category relation network to extend information in both user and item domains. This enables the joint construction of a CHIN, which is input into the embedding layer to obtain user, item, and category node embeddings. These embeddings are then fed into CoHet4Rec for collaborative heterogeneous information propagation, enhanced by meta-path adaptive heterogeneous relation attention and collaborative heterogeneous neighbor information aggregation.

We introduce the CoHet4Rec framework (Fig 2), which comprises three essential modules: the embedding layer, the collaborative heterogeneous neighbor attention layer, and the prediction and optimization stage. The embedding layer embeds the features of nodes, the collaborative heterogeneous neighbor attention layer mainly updates the features of nodes, and the prediction and optimization stage mainly trains and optimizes the model.

### Embedding layer

To preserve social connections, user-item interaction, item-category relationships and their rich semantic information, we combine the interaction, social and relation sub-graph into a unified CHIN *G*.


$$\begin{array}{ccc} G=(N,E), & & \end{array}$$ (1)

where *N* = *U* ∪ *V* ∪ *C* is the set of user/item/category nodes, and *E* = *X* ∪ *S* ∪ *T* denotes the set of edges connecting these nodes.

We maintain an embedding layer $H\in {\mathbb{R}}^{(N+M+K)\times d}$, wherein each row corresponds to a learnable node representation mapping parameter. By indexing the layer, we can obtain the embedding $H_{v}\in {\mathbb{R}}^{d}$ for a given node *v* ∈ *N*.

### Collaborative heterogeneous neighbor attention layer

The CoHet4Rec framework utilizes a CHIN *G* as the input computational graph, which encompasses three types of heterogeneous relations (or can be extended to include multiple relations) for collaborative heterogeneous information propagation (as depicted in the left portion of Fig 2). The attention layer primarily encompasses two steps: collaborative heterogeneous relation information propagation with attention enhancement and multi-heterogeneous collaborative relation information aggregation.

We can formally represent the processes of information propagation and aggregation at two distinct levels (from layer  (ℓ) to layer  (ℓ+1)):

**Edge-wise:**


$$\begin{array}{ccc} {msg}_{\left(u,v\right)}^{\left(\ell +1\right)}=\phi \left(H_{u}^{\left(\ell \right)},H_{v}^{\left(\ell \right)},Atn_{\left(u,v\right)}^{\left(\ell \right)}\right),(u,v)\in E, & & \end{array}$$ (2)

where *ϕ* ( ⋅ )  is the message function used to propagate messages between node *u* and *v* along the connecting edge  ( *u* , *v* )  according to the attention weights $Atn_{\left(u,v\right)}^{\left(\ell \right)}$ of the heterogeneous relations.

**Node-wise:**


$$\begin{array}{ccc} H_{u}^{\left(\ell +1\right)}=\psi \left(H_{u}^{\left(\ell \right)},\underset{\forall v\in N(u)}{Agg}\left(\left\{{msg}_{\left(u,v\right)}^{\left(\ell +1\right)}:(u,v)\in E\right\}\right)\right), & & \end{array}$$ (3)

where *ψ* ( ⋅ )  is the message update function used to update the own message of *u*. $H_{u}^{\left(\ell \right)}$ represents *u*’s embedding at the  (ℓ)-th layer. *N* ( *u* )  represents the set of 1^st^-order neighbors of *u*. The function *Agg* ( ⋅ )  serves as a message aggregation mechanism, responsible for combining the messages received from the 1^st^-order neighboring nodes.

**Specifically:** (1) To capture the enhanced collaborative heterogeneous features, which are influenced by the knowledge of user domain and item domain during the process of collaborative heterogeneous information propagation with attention enhancement, we employ external heterogeneous relation attention units to parameterize the heterogeneity and collaboration of relations as embedding projections. As a result, the graph encoder is capable of acquiring meaningful non-linear characteristics from collaborative heterogeneous relation representations. In order to maintain the collaborative semantics among different nodes and edges, a shared hyper-parameter space is utilized to encode the collaborative heterogeneous relations with heterogeneous relation attention. This approach enables us to learn collaborative semantic features that capture various potential collaborative influencing factors within the graph *G*. Specifically, the heterogeneous relation attention encoder is given by Eqs (4) and (5):


$$\begin{array}{ccc} \phi \left(H_{u}^{\left(\ell \right)},H_{v}^{\left(\ell \right)},Atn_{\left(u,v\right)}^{\left(\ell \right)}\right)=\left(Atn_{\left(u,v\right)}^{\left(\ell \right)}\right)H_{u}^{\left(\ell \right)}, & & \end{array}$$ (4)

$$\begin{array}{ccc} Atn_{\left(u,v\right)}^{\left(\ell \right)}=\text{Linear}1\left(\text{LeakyReLU}\left(\text{Linear}2\left(H_{v}^{\left(\ell \right)}\right)\right)\right), & & \end{array}$$ (5)

where, since the representations learned by nodes from different relationships are heterogeneous, we employ a Multi-Layer Perceptron (MLP) based attention calculation network to learn the heterogeneous relationships between nodes and project these representations into the same semantic space. The trainable transformation matrix in *Linear*1 ( ⋅ )  is represented as $W^{1}\in {\mathbb{R}}^{d\times d}$ and is represented as $W^{2}\in {\mathbb{R}}^{d}$ in *Linear*2 ( ⋅ ) . The trainable bias terms in *Linear*2 ( ⋅ )  is represented as $b^{2}\in \mathbb{R}$. The negative slope is set to 0.2 for better gradient back-propagation. *u* is the source node and *v* is the target node.

**Fig 3:**
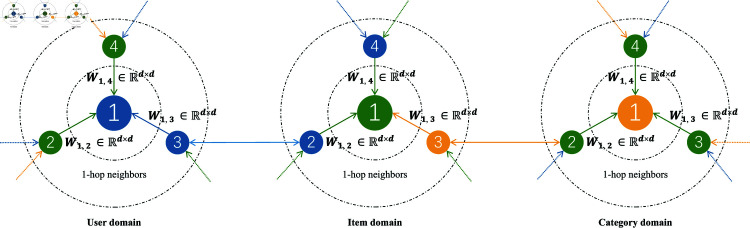
High-order information propagation of CoHet4Rec.

(2) Regarding the aggregation of multi-heterogeneous collaborative relation information: after conducting collaborative heterogeneous information propagation with attention enhancement, the subsequent step involves aggregating the heterogeneous relation semantics from the 1^st^-order collaborative heterogeneous neighbors of the nodes. We need to aggregate messages from various sources of information, such as users, items, and categories (as depicted in Fig 3). For instance, in the case of node (which can represent a user node, item node, or category node), we combine the messages from the 1^st^-order heterogeneous relation context in the message aggregator, as represented by Eq (6). After multiple information propagation and aggregation processes, the model can obtain high-order information of nodes in the same domain and different domains (as shown by the unidirectional dashed line and bidirectional solid line in Fig 3).


$$\begin{array}{ccc} H_{u}^{\left(\ell +1\right)}=\frac{\underset{\forall v\in N(u)}{\sum }\phi \left(H_{u}^{\left(\ell \right)},H_{v}^{\left(\ell \right)},Atn_{\left(u,v\right)}^{\left(\ell \right)}\right)}{\left|N(u)\right|}, & & \end{array}$$ (6)

where  | *N* ( *u* ) |  represents the number of 1^st^-order collaborative heterogeneous neighbors of a given node *u*.

To ensure stable network training, following [37,40], we also utilize a combination of self-propagation and layer normalization techniques. This helps to enhance the overall stability of the training process. In terms of self-loop, we treats the self node as a neighbor and applies attention encoding.


$$\begin{array}{ccc} {\tilde{H}}_{u}^{\left(\ell +1\right)}=\frac{\alpha *\left(H_{u}^{\left(\ell +1\right)}-\mu \right)}{\sigma +eps}+\beta +\psi \left(H_{u}^{\left(\ell \right)},H_{u}^{\left(\ell \right)}\right), & & \end{array}$$ (7)

where, we introduce learnable scaling factors *α* and bias terms *β*. *μ* and *σ* are the mean and standard deviation of $H_{u}^{\left(\ell +1\right)}$, respectively. *eps* is a small constant.

We also conducted cross-layer residual feature aggregation (as depicted in Eq (8) and the left portion of Fig 2) to fully leverage multi-layer node embedding.


$$\begin{array}{ccc} H_{u}^{*}=\text{LayerNorm}\left({\tilde{H}}_{u}^{\left(0\right)}|{\tilde{H}}_{u}^{\left(1\right)}|\cdots |{\tilde{H}}_{u}^{\left(L\right)}\right), & & \end{array}$$ (8)

where $H_{u}^{*}\in {\mathbb{R}}^{d}$ represents the final node embedding. ‘ ∣ ’ represents embedding concatenation.

### Prediction and optimization

During the prediction phase, we calculate user preferences for items by calculating the inner product of user and item features. Our optimization objective is defined by Eq (9), which includes a composite loss: the pairwise Bayesian Personalized Ranking (BPR) loss and a regularization term for weight decay.


$$\begin{array}{ccc} L_{BPR}=-\sum log\left(Sigmoid\left(x_{u_{ij}}\right)\right)+\lambda ∥\theta {∥}^{2}, & & \end{array}$$ (9)

$$\begin{array}{ccc} x_{u_{ij}}=\left(H_{u}^{{*}^{T}}\cdot H_{v_{i}}^{*}\right)-\left(H_{u}^{{*}^{T}}\cdot H_{v_{j}}^{*}\right), & & \end{array}$$ (10)

where $x_{u_{ij}}$ represents the difference in preference between the model’s predictions for positive sample item *i* and negative sample item *j* for user *u*. *λ* represents the weight of the regularization term to avoid over-fitting, *θ* represents the training parameters. $H_{v_{i}}^{*}$ and $H_{v_{j}}^{*}$ represent the embeddings of the positive and negative item *i* and *j*, respectively. To maximize $Sigmoid\left(x_{u_{ij}}\right)$, which represents the probability that the user’s preference for the positive sample is higher than the negative one, is the optimization objective.

During the optimization phase, we iteratively update *θ* by gradient descent (Eq 11).


$$\begin{array}{ccc} \theta =\theta -\eta \frac{\partial L_{BPR}}{\partial \theta }, & & \end{array}$$ (11)

where *η* is the learning rate. The process of CoHet4Rec is detailed in Algorithm 1.

**Algorithm 1**. CoHet4Rec



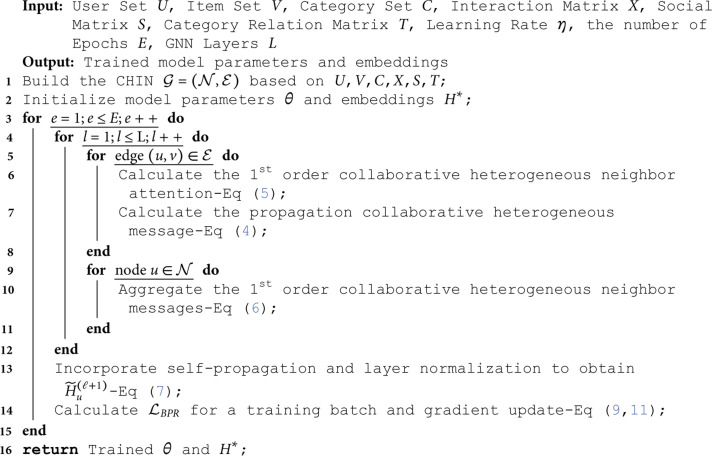



## Results

We provide the outcomes of extensive experiments performed to evaluate the efficacy of CoHet4Rec, employing three openly accessible datasets. We proposed several research questions (RQs) to evaluate the performance of our method:

- **RQ1**: Is CoHet4Rec better than existing methods?
- **RQ2**: Are these components necessary in CoHet4Rec?
- **RQ3**: Are these CHINs important?
- **RQ4**: How does data sparsity affect model performance?
- **RQ5**: How efficient is the model?
- **RQ6**: What is the impact of hyper-parameters?

RQ1 section conducted a comparative study on the performance of the model and roughly analyzed several important contributions to the success of the model; Then, detailed experimental verification and analysis were conducted on these contributions in RQ2 and RQ3 section, respectively. In addition, RQ4 and RQ5 section studied the robustness of the model to data sparsity and time cost. Finally, RQ6 section explored the influence of some important hyper-parameters that affect model performance.

### Experimental setup

**Datasets:** In this paper, we performed experiments and analysis using three widely-used social RS datasets (Ciao, Epinions and Yelp) which were collected from various online review systems in real-world. Ciao and Epinions are available at https://www.cse.msu.edu/ tangjili/ datasetcode/truststudy.htm, Yelp is available at https://www.yelp.com/dataset. Table 1 provides comprehensive statistical information about these datasets.

**Table 1:** Statistics of datasets.

Datasets	Ciao	Epinions	Yelp
Users	1925	18081	99262
Items	15053	251722	105142
Categories	6	28	1336
# of user-item interactions	30370	715821	769929
Interaction density	0.1048%	0.0157%	0.0074%
# of social connections	65084	572784	1298522
Social connection density	1.7564%	0.1752%	0.0132%
# of item-category connections	15056	252317	464486
Item-category connection density	16.6700%	3.5799%	0.3307%

**Baselines:** Following [37], we comprehensively evaluate the performance of CoHet4Rec with 15 baselines from 7 different types:

**Graph-CF RS**

- GCCF [41]: A linear residual GCN-based CF recommendation model. It aims to solve the problems of GCN CF models in terms of training difficulties, nonlinear activation functions, and deep stacking.
- NGCF [42]: This CF model utilizes graph convolution to leverage the user-item graph structure. It effectively represents and captures high-order connectivity relationships by propagating features on the graph.

**GNN-based RS**

- GraphRec [8]: A GNN-based social RS algorithm that can simultaneously consider the influence of interaction and related viewpoints.
- DiffNet [43]: Utilizes graph information propagation to establish a hierarchical diffusion architecture for modeling users’ social relationships. It simulates dynamic social diffusion to capture recursive social influence.
- MHCN [31]: A hypergraph convolutional networks-based social RS that utilizes high-order user relationships to improve recommendation quality.

**Disentangled graph RS**

- DGNN [37]: It explores social recommendation by learning disentangled heterogeneous factors and improves social recommendation by disentangling the heterogeneous relationships between users and item domains.
- DGCF [24]: It focuses particularly on user-item relationships with a finer granularity of user intent and models the intention distribution of each user-item interaction and iteratively refines the intention aware interaction graph and representation.
- DisenHAN [36]: A disentangled heterogeneous GAT for Top-K recommendation. It learns user/item representations for disentanglement in HINs from different perspectives.

**Time-aware social RS**

- DGRec [44]: It models dynamic user behavior using RNN and contextual social impact using GAT.

**Knowledge-aware RS**

- KGAT [25]: This model employs attention mechanisms to aggregate information from both interactions and the item knowledge.

**Attention-based social RS**

- SAMN [30]: In this model, a two-stage attention mechanism is employed to capture user influence and identify relevant friends for preference modeling. It introduces a friend-style attention to explore the social influence between users.
- EATNN [45]: It utilizes attention mechanisms to integrate interactive and social information. It combines an optimization scheme to implement a multitasking learning framework.

**Heterogeneous graph embedding-based RS**

- HERec [15]: A HIN embedding-based method. It learns node embeddings by generating meaningful node sequences through a random walk strategy based on meta-path.
- HGT [14]: In this method, specific parameters are designed to capture the heterogeneous attention on each edge, taking into account the node and edge types. This enables the model to maintain distinct embeddings for various types of nodes and edges.
- HAN [27]: A attention mechanism-based heterogeneous GNN method. It considers the diversity of nodes and edges in heterogeneous networks, adopts attention mechanisms at both node and semantic levels, and generates node embedding representations through hierarchical aggregation of node features.

**Evaluation Metrics:** In our study, CoHet4Rec focuses on providing recommendations of the Top-K items for users. To evaluate and compare the models, we use two widely adopted metrics in RS: Hits Ratio (HR@K) and Normalized Discounted Cumulative Gain (NDCG@K) [13]. HR@K and NDCG@K provide performance evaluations of RS in the face of data sparsity and cold-start problem, helping to determine whether the model can still provide effective recommendations under these challenges.

HR@K measures the proportion of correctly recommended items in the Top-K list that match the user’s actual interactions (positive items). It emphasizes accuracy, ranging from 0 to 1, with higher values indicating better performance. NDCG@K evaluates the balance between item relevance and ranking within the Top-K recommendation list, focusing on the orderliness of the predicted results. Its numerical range and significance are consistent with HR@K.

To compute HR@K and NDCG@K, we choose 100 items as negative samples (i.e., items that the user has not interacted with) and combine them with positive instances. By evaluating these combinations, we can evaluate the model’s performance based on the metrics. The calculations and interpretations of these metrics are as follows:


$$\begin{array}{ccc} HR@K=\frac{1}{N}\overset{N}{\underset{i=1}{\sum }}\overset{K}{\underset{j=1}{\sum }}hits(i,j), & & \end{array}$$ (12)

where, *N* denotes the number of test users. The variable *hits* ( *i* , *j* )  represents whether the item *j* predicted by the model for the user *i* is positive. Specifically, *hits* ( *i* , *j* )  takes the value 1 if the item is positive and 0 otherwise.


$$\begin{array}{ccc} NDCG@K=\frac{1}{N}\overset{N}{\underset{i=1}{\sum }}\frac{DCG_{i}@K}{IDCG_{i}@K}, & & \end{array}$$ (13)

$$\begin{array}{ccc} DCG_{i}@K=\overset{K}{\underset{j=1}{\sum }}\frac{2^{hits(i,j)}-1}{log_{2}(j+1)}, & & \end{array}$$ (14)

where the Discounted Cumulative Gain (DCG@K) quantifies the relevance of the items and their positions within the recommendation list. It evaluates the balance between item relevance and ranking order among the Top-K recommended items. IDCG@K refers to the Ideal situation (the possible maximum value) of DCG@K.

**Parameter Settings:** When adjusting model parameters, we defined the following ranges: GNN layers (L):  { 1 , 2 , 3 , 4 } , regularization coefficient (*λ*):  { 0 . 005 , 0 . 0005 , 0 . 00005 } , embedding dimension (d):  { 4 , 8 , 16 } , and heterogeneous relation attention encoding hidden dimension (H):  { 2 , 4 , 8 , 16 } . To ensure robustness, we performed 10 iterations of all tasks and present the average results.

### RQ1-Performance evaluation

We conducted a comprehensive comparison of various baselines on three datasets. The main results are presented in Table 2. Based on the experimental findings, it is evident that CoHet4Rec surpasses the current SOTA models in predicting the Top-5 recommended items. Compared to the worst-performing model, CoHet4Rec achieved average relative improvements of 31.88% and 38.39% in HR@5 and NDCG@5, respectively. Furthermore, compared to the second-best model, we achieved average relative improvements of 3.19% in HR@5 and 3.81% in NDCG@5.

**Table 2:** The performance of various models was compared using HR@K and NDCG@K metrics.

Datasets	Method	HR @5	Imp	NDCG @5	Imp	HR @10	Imp	NDCG @10	Imp	HR @20	Imp	NDCG @20	Imp
**Ciao**	**GCCF** **NGCF** **GraphRec** **DiffNet** **MHCN** **DGNN** **DGCF** **DisenHAN** **DGRec** **KGAT** **SAMN** **EATNN** **HERec** **HGT** **HAN** **Ours**	0.3685 0.3570 0.3058 0.3941 0.3864 0.4120 0.3871 0.3493 0.3724 0.3391 0.3468 0.2969 0.3832 0.3415 0.2937 **0.4402**	**19.46%** **23.31%** **43.95%** **11.70%** **13.92%** **6.84%** **13.72%** **26.02%** **18.21%** **29.81%** **26.93%** **48.27%** **14.87%** **28.90%** **49.88%** **—**	0.2668 0.2360 0.2235 0.2816 0.2799 0.2890 0.2782 0.2482 0.2647 0.2422 0.2460 0.2124 0.2679 0.2372 0.1897 **0.3115**	**16.75%** **31.99%** **39.37%** **10.62%** **11.29%** **7.79%** **11.97%** **25.50%** **17.68%** **28.61%** **26.63%** **46.66%** **16.27%** **31.32%** **64.21%** **—**	0.4926 0.4843 0.4594 0.5202 0.5080 0.5515 0.5189 0.4856 0.5086 0.4907 0.4677 0.4130 0.5298 0.4933 0.4856 **0.5733**	**16.38%** **18.38%** **24.79%** **10.21%** **12.85%** **3.95%** **10.48%** **18.06%** **12.72%** **16.83%** **22.58%** **38.81%** **8.21%** **16.22%** **18.06%** **—**	0.3070 0.3088 0.2670 0.3201 0.3118 0.3338 0.3166 0.2894 0.3113 0.2977 0.2838 0.2520 0.3104 0.3062 0.2608 **0.3535**	**15.15%** **14.48%** **32.40%** **10.43%** **13.37%** **5.90%** **11.66%** **22.15%** **13.56%** **18.74%** **24.56%** **40.28%** **13.89%** **15.45%** **35.54%** **—**	0.6289 0.5937 0.5976 0.6647 0.6321 0.6942 0.6775 0.6161 0.6219 0.6052 0.6251 0.5222 0.6846 0.6128 0.6513 **0.7274**	**15.66%** **22.52%** **21.72%** **9.43%** **15.08%** **4.78%** **7.37%** **18.07%** **16.96%** **20.19%** **16.37%** **39.30%** **6.25%** **18.70%** **11.68%** **—**	0.3141 0.3118 0.3042 0.3573 0.3453 0.3726 0.3604 0.3248 0.3227 0.3326 0.3223 0.2819 0.3641 0.3229 0.2821 **0.3920**	**24.80%** **25.72%** **28.86%** **9.71%** **13.52%** **5.21%** **8.77%** **20.69%** **21.48%** **17.86%** **21.63%** **39.06%** **7.66%** **21.40%** **38.96%** **—**
**Epinions**	**GCCF** **NGCF** **GraphRec** **DiffNet** **MHCN** **DGNN** **DGCF** **DisenHAN** **DGRec** **KGAT** **SAMN** **EATNN** **HERec** **HGT** **HAN** **Ours**	0.5538 0.5612 0.5683 0.5106 0.5199 0.6142 0.5479 0.5609 0.5053 0.5483 0.5176 0.5283 0.5519 0.5757 0.5403 **0.6232**	**12.53%** **11.05%** **9.66%** **22.05%** **19.87%** **1.47%** **13.74%** **11.11%** **23.33%** **13.66%** **20.40%** **17.96%** **12.92%** **8.25%** **15.34%** **—**	0.4161 0.4316 0.4325 0.3820 0.3883 0.4794 0.4144 0.4247 0.3775 0.4139 0.3860 0.3924 0.4179 0.4360 0.4106 **0.4873**	**17.11%** **12.91%** **12.67%** **27.57%** **25.50%** **1.65%** **17.59%** **14.74%** **29.09%** **17.73%** **26.24%** **24.18%** **16.61%** **11.77%** **18.68%** **—**	0.6779 0.6944 0.6865 0.6323 0.6411 0.7335 0.6635 0.6825 0.6268 0.6756 0.6390 0.6422 0.6767 0.7001 0.6673 **0.7365**	**8.64%** **6.06%** **7.28%** **16.48%** **14.88%** **0.41%** **11.00%** **7.91%** **17.50%** **9.01%** **15.26%** **14.68%** **8.84%** **5.20%** **10.37%** **—**	0.4783 0.4763 0.4786 0.4160 0.4261 0.5215 0.4594 0.4627 0.4127 0.4708 0.4259 0.4483 0.4572 0.4812 0.4371 **0.5239**	**9.53%** **9.99%** **9.47%** **25.94%** **22.95%** **0.46%** **14.04%** **13.23%** **26.94%** **11.28%** **23.01%** **16.86%** **14.59%** **8.87%** **19.86%** **—**	0.7906 0.8010 0.8001 0.7367 0.7496 0.8281 0.7770 0.7890 0.7308 0.788 0.7491 0.7501 0.7792 0.8053 0.7761 **0.8368**	**5.84%** **4.47%** **4.59%** **13.59%** **11.63%** **1.05%** **7.70%** **6.06%** **14.50%** **6.19%** **11.71%** **11.56%** **7.39%** **3.91%** **7.82%** **—**	0.4852 0.5006 0.5011 0.4476 0.4551 0.5387 0.4811 0.4911 0.4429 0.4837 0.4553 0.4557 0.4839 0.5029 0.4802 **0.5498**	**13.31%** **9.83%** **9.72%** **22.83%** **20.81%** **2.06%** **14.28%** **11.95%** **24.14%** **13.67%** **20.76%** **20.65%** **13.62%** **9.33%** **14.49%** **—**
**Yelp**	**GCCF** **NGCF** **GraphRec** **DiffNet** **MHCN** **DGNN** **DGCF** **DisenHAN** **DGRec** **KGAT** **SAMN** **EATNN** **HERec** **HGT** **HAN** **Ours**	0.6703 0.6748 0.6631 0.6701 0.6607 0.7052 0.6565 0.6511 0.6511 0.6503 0.6359 0.6425 0.5833 0.6888 0.6635 **0.7141**	**6.53%** **5.82%** **7.69%** **6.57%** **8.08%** **1.26%** **8.77%** **9.68%** **9.68%** **9.81%** **12.30%** **11.14%** **22.42%** **3.67%** **7.63%** **—**	0.5130 0.5192 0.4903 0.5127 0.4911 0.5378 0.4958 0.4944 0.4897 0.4901 0.4662 0.4866 0.4501 0.5136 0.5080 **0.5485**	**6.92%** **5.64%** **11.87%** **6.98%** **11.69%** **1.99%** **10.63%** **10.94%** **12.01%** **11.92%** **17.65%** **12.72%** **21.86%** **6.80%** **7.97%** **—**	0.8130 0.8204 0.8019 0.8222 0.8019 0.8373 0.7956 0.8159 0.7830 0.7737 0.7971 0.7273 0.7047 0.8185 0.8169 **0.8393**	**3.23%** **2.30%** **4.66%** **2.08%** **4.66%** **0.24%** **5.49%** **2.87%** **7.19%** **8.48%** **5.29%** **15.40%** **19.10%** **2.54%** **2.74%** **—**	0.5585 0.5651 0.5372 0.5524 0.5348 0.5873 0.5410 0.5403 0.5386 0.5386 0.5293 0.5289 0.4990 0.5547 0.5511 **0.5892**	**5.50%** **4.26%** **9.68%** **6.66%** **10.17%** **0.32%** **8.91%** **9.05%** **9.39%** **9.39%** **11.32%** **11.40%** **18.08%** **6.22%** **6.91%** **—**	0.9011 0.9011 0.8944 0.9053 0.8958 0.9293 0.9010 0.9040 0.8824 0.8795 0.9009 0.8066 0.8125 0.9060 0.8977 **0.9369**	**3.97%** **3.97%** **4.75%** **3.49%** **4.59%** **0.82%** **3.98%** **3.64%** **6.18%** **6.53%** **4.00%** **16.15%** **15.31%** **3.41%** **4.37%** **—**	0.5503 0.5684 0.5650 0.5701 0.5670 0.6043 0.5678 0.5650 0.5611 0.5521 0.5407 0.5468 0.5034 0.5802 0.5529 **0.6103**	**10.90%** **7.37%** **8.02%** **7.05%** **7.64%** **0.99%** **7.49%** **8.02%** **8.77%** **10.54%** **12.87%** **11.61%** **21.24%** **5.19%** **10.38%** **—**

**Note:** Top-performing results are underlined. The relative performance improvement of our model compared to the baseline is denoted as ‘Imp’.

The advantages of CoHet4Rec are supported by:

(1) **Enhanced Interaction Learning:** CoHet4Rec incorporates social and item-category relationship information, enhancing the model’s capability to learn interactions between users and items, which improves recommendation performance. Comparative analysis with models focusing primarily on social recommendations confirms the performance enhancement of CoHet4Rec.

(2) **Effective Semantic Integration:** The heterogeneous relation attention and aggregation of HIN enable effective integration of semantic information from interactions, social connections, and item-category relationships. This facilitates better modeling of user and item features. Compared to disentangled graph RS, CoHet4Rec employs a specifically designed heterogeneous relation attention-enhanced network structure to capture the relevance between users and items, leading to improved performance.

GNN-based social recommendation models outperform attention-based solutions, showcasing the effectiveness of using a multi-hop graph structure for embedding propagation to model social relationships. CoHet4Rec, with its designed heterogeneous relation attention-enhanced network structure, can learn potential connections by considering multi-hop neighbors from user and item domains.

Table 2 also shows significant performance improvements of CoHet4Rec compared to other competing models in Top-K (K  =  10, 20) recommendation scenarios. This further validates the exceptional ranking capabilities of our framework. As K increases, we also observe an improvement in recommendation accuracy.

### RQ2-Component ablation study

We created three variants to analyze the roles of key components in CoHet4Rec and verify their efficacy:

- **w/o-GNN:** This variant removes the GNN layers used by CoHet4Rec to propagate information among nodes.
- **w/o-Attention:** This variant eliminates the heterogeneous collaborative neighbor attention used by CoHet4Rec to learn weights for the heterogeneous relationships among user/item/category neighbors.
- **w/o-LN:** This variant omits the layer normalization applied in each propagation layer by CoHet4Rec.

The performance of various methods, including CoHet4Rec and its variants, is presented in Table 3 across three datasets. we draw the following conclusions and insights:

**Table 3:** Component ablation study.

Variant	Ciao@5		Epinions@5		Yelp@5	
	HR	NDCG	HR	NDCG	HR	NDCG
**w/o-GNN** **Relative difference**	**0.1798** 144.83%	**0.1187** 162.43%	**0.4900** 27.18%	**0.3645** 33.69%	**0.3870** 84.52%	**0.2733** 100.70%
**w/o-Attention** **Relative difference**	**0.3980** 10.60%	**0.2824** 10.30%	**0.6075** 2.58%	**0.4747** 2.65%	**0.7121** 0.28%	**0.5464** 0.38%
**w/o-LN** **Relative difference**	**0.4296** 2.47%	**0.3050** 2.13%	**0.6095** 2.25%	**0.4742** 2.76%	**0.7061** 1.13%	**0.5381** 1.93%
**CoHet4Rec**	**0.4402**	**0.3115**	**0.6232**	**0.4873**	**0.7141**	**0.5485**

**Note:** Top-performing results are underlined. ‘Relative difference’ indicates the performance difference between each variant and CoHet4Rec.

(1) **Superior Performance of CoHet4Rec: ** CoHet4Rec consistently outperforms its three variants, emerging as the top-performing method. The ‘w/o-GNN’ variant shows the weakest performance, highlighting the significant contribution of the GNN architecture to the model’s effectiveness. This underscores the importance of incorporating GNN in CoHet4Rec for superior performance.

(2) **Importance of Attention Mechanism: ** The performance of the ‘w/o-Attention’ variant declines compared to CoHet4Rec, indicating the crucial role of the heterogeneous relationship-enhanced attention encoder in exploring potential collaborative relationships between neighbor nodes.

(3) **Role of Layer Normalization: ** Comparing with the ‘w/o-LN’ variant, it is evident that layer normalization is vital in the training process. It stabilizes gradient generation during model training, prevents premature over-smoothing in GNN, and thus significantly impacts the model’s performance.

### RQ3-CHIN ablation study

To explore the influence of various auxiliary relationships on CoHet4Rec’s performance, we examined three variants of auxiliary relationships:

- **w/o-S:** Social matrix *S* is removed from CHIN.
- **w/o-T:** Item-category relationship matrix *T* is removed.
- **w/o-ST:** Both *S* and *T* are removed.

**Fig 4:**
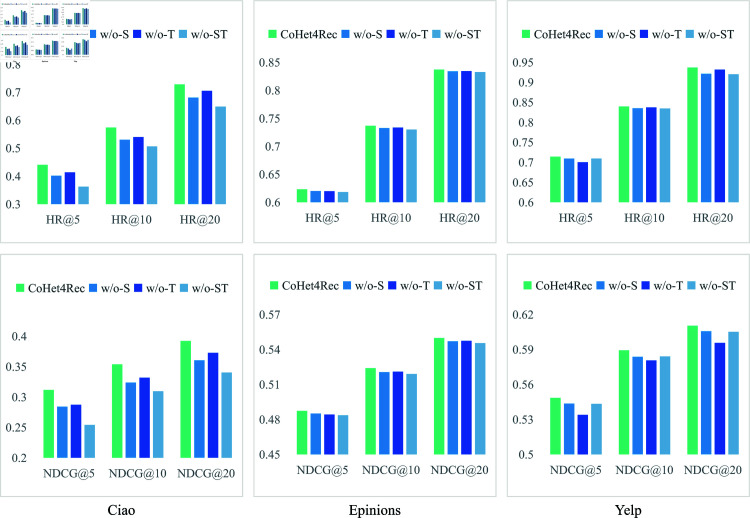
The influence of various CHIN on the performance of CoHet4Rec across three datasets.

We evaluated the performance on three datasets with varying Top-K settings (Fig 4) and derived the following key findings and conclusions:

(1) **Positive impact of auxiliary relationships:** Adding auxiliary heterogeneous relationships consistently improves model performance. This may be due to the benefits of integrating multiple heterogeneous semantics into the representation, allowing CoHet4Rec to explore collaborative relationships within diverse CHIN.

(2) **Dataset-specific observations:** In Ciao and Epinions, the ‘w/o-T’ variant performs sub-optimally, highlighting the benefits of utilizing social context signals for user preference learning. In Yelp, however, ‘w/o-T’ performs the worst in most cases, even lower than ‘w/o-ST’, indicating some inconsistencies.

(3) ** Worst performance of ‘w/o-ST’:** The ‘w/o-ST’ variant consistently performs the worst in most cases, reinforcing that combining heterogeneous relationships from the user/item domain enhances recommendation accuracy. However, similar inconsistencies are observed in Yelp as noted in the second point.

The inconsistencies mentioned in points (2) and (3) may be due to the varying impact of alleviating data sparsity in collaborative relationships across datasets. Adding too many collaborative relationships might introduce unnecessary noise. For example, while we assume a relationship between user interests and social connections, ConsisRec [28] highlights the often overlooked aspect of social inconsistency in existing methods. Specifically, social inconsistency indicates that the interests of users with social connections may not necessarily align. Aggregating information from inconsistent social neighbors together can undermine the ability of GNN to describe information that is beneficial for recommendation. This suggests that social relationships alone may not be sufficient for accurately predicting user ratings, and other factors need to be considered in the recommendation process. Similarly, item-category relationships may not always be relevant for link prediction.

### RQ4-Robustness in sparse data scenarios

**Fig 5:**
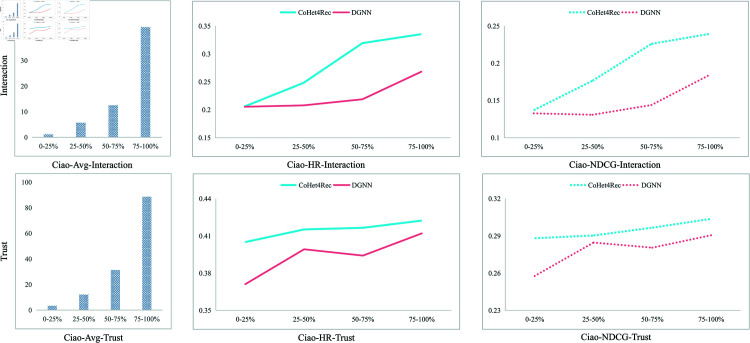
Model performance under sparse data.

To investigate the robustness of CoHet4Rec in sparse data scenarios, we first ranked all users by their interaction density and social connection density in ascending order. Subsequently, we divided them into quartiles (0–25%, 25–50%, 50–75%, and 75–100%), each containing an equal number of users. Fig 5 depicts the average interactions and social connection count for each user group.

It is evident that CoHet4Rec performs better than the baseline in different types of sparse data scenarios, indicating the robustness of our model in handling sparse user behavior data. Coupled with the results in Table 2, it is clear that our model surpasses some SOTA models even under sparse data conditions. This further confirms the effectiveness of CoHet4Rec in mitigating cold-start problem by leveraging external knowledge from both user and item domains in situations of data scarcity.

### RQ5-Model efficiency study

**Fig 6:**
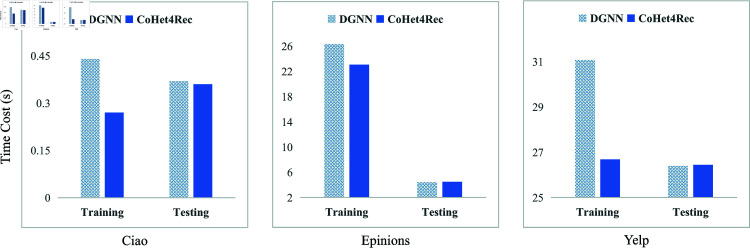
Time cost statistics for model training and testing phases.

We assessed the models’ time cost from two perspectives: average training and testing runtime. The experiments compared our proposed CoHet4Rec model with the SOTA baseline DGNN [37]. Fig 6 shows the average training/testing runtime per epoch.

From the results, we observed that our non-disentangled CHIN algorithm achieves faster model training with nearly identical testing time compared to the disentangled RS DGNN. Specifically, the average training time was reduced by 21.74% across three datasets. This reduction is due to DGNN’s decoupled hierarchical message propagation approach with multiple relationships, which leads to time-consuming message propagation. Additionally, larger *G* results in longer training times.

### RQ6-Parameter selection

**Fig 7:**
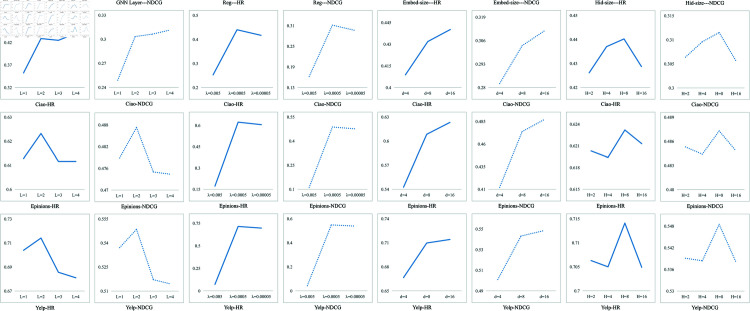
Results of hyper-parameter ablation experiments on three datasets.

We investigated the hyper-parameters affecting CoHet4Rec’s performance (Fig 7). By analyzing these relationships, we gain valuable insights into how hyper-parameter selection impacts CoHet4Rec’s performance, guiding effective tuning and optimization of the model’s parameters.

**GNN layers (L):** Fig 7 indicates that propagating embeddings between 2-hop neighbor nodes improves performance, highlighting the efficacy of encoding higher-order relationships. However, stacking more layers leads to performance degradation due to the over-smoothing problem.

**Regularization coefficient (*λ*):** Adding a regularization term in the layer normalization process helps prevent over-fitting. The performance decreases when *λ* is excessively large or small, emphasizing the importance of selecting an appropriate *λ* range to optimize performance.

**Embedding dimension (d):** The model performs best when d=16. Performance deteriorates as the d increases. Our model adopts smaller d while maintaining high performance, facilitating deployment in real-world scenarios.

**Hidden dimension of heterogeneous relationship attention encoding (H):** CoHet4Rec captures the semantics between multiple heterogeneous connections by exploring collaborative factors among heterogeneous relationships. When H=8, the performance is optimal.

## Conclusion and future work

This paper presents CoHet4Rec, a novel social RS that leverages GNNs and CHIN. By integrating heterogeneous relationship attention units into the GNN framework, CoHet4Rec effectively captures and utilizes collaborative relationships from both the user and item domains. Extensive comparative and ablation experiments show the effectiveness of CoHet4Rec. Although CoHet4Rec demonstrates commendable performance in handling multiple collaborative relationships, deeper exploration is still needed for fine-grained processing of these relationships, such as introducing item-item relationships and more detailed forms of user-item interactions (e.g., different ratings). Additionally, exploring new neighbor sampling based message propagation and aggregation schemes will further reduce the training time of the model, which is crucial for practical deployment. Finally, further extending the model to federated learning will help in protecting user privacy.

## References

[1] Zhou Z, Zhang L, Yang N. Contrastive collaborative filtering for cold-start item recommendation. In: 2023 Proceedings of the ACM web conference. 2023. p. 928–937.

[2] Jafri SIH, Ghazali R, Javid I, Mahmood Z, Hassan AAA. Deep transfer learning with multimodal embedding to tackle cold-start and sparsity issues in recommendation system. PLoS One 2022;17(8):e0273486. doi: 10.1371/journal.pone.0273486 36007091 PMC9410545

[3] Liu Z, Meng L, Jiang F, Zhang J, Yu PS. Deoscillated graph collaborative filtering. arXiv Preprint. 2020. doi: arXiv:201102100

[4] Huang F, Wang Z, Huang X, Qian Y, Li Z, Chen H. Aligning distillation for cold-start item recommendation. In: Proceedings of the 46th international ACM SIGIR conference on research and development in information retrieval. 2023. p. 1147–1157.

[5] Ma H, Yang H, Lyu MR, King I. Sorec: social recommendation using probabilistic matrix factorization. In: Proceedings of the 17th ACM conference on information and knowledge management. 2008. p. 931–940.

[6] Wang X, Pan W, Xu C. HGMF: Hierarchical group matrix factorization for collaborative recommendation. In: Proceedings of the 23rd ACM international conference on conference on information and knowledge management. 2014. p. 769–778.

[7] Ye F, Lu X, Li H, Chen Z. Transfer learning from rating prediction to Top-k recommendation. PLoS One 2024;19(3):e0300240. doi: 10.1371/journal.pone.0300240 38547150 PMC10977712

[8] Fan W, Ma Y, Li Q, He Y, Zhao E, Tang J, et al. Graph neural networks for social recommendation. In: Proceedings of the world wide web conference. 2019. p. 417–426.

[9] Hu C, Fan W, Zeng E, Hang Z, Wang F, Qi L, et al. Digital twin-assisted real-time traffic data prediction method for 5G-enabled internet of vehicles. IEEE Trans Industr Inform. 2021;18(4):2811–19.

[10] Bizzi L, Labban A. The double-edged impact of social media on online trading: Opportunities, threats, and recommendations for organizations. Bus Horiz. 2019;62(4):509–19.

[11] Fan W, Ma Y, Li Q, Wang J, Cai G, Tang J. A graph neural network framework for social recommendations. IEEE Trans Knowl Data Eng. 2020;34(5):2033–47.

[12] Sharma K, Lee YC, Nambi S, Salian A, Shah S, Kim SW, et al. A survey of graph neural networks for social recommender systems. ACM Comput Surv. 2024;56(10):1–34.

[13] Li N, Guo B, Liu Y, Yu Z. Disentangled-feature and composite-prior VAE on social recommendation for new users. Expert Syst Appl. 2024;247:123309.

[14] Hu Z, Dong Y, Wang K, Sun Y. Heterogeneous graph transformer. In: 2020 Proceedings of the web conference. 2020. p. 2704–2710. doi: 10.1145/3366423.3380027

[15] Shi C, Hu B, Zhao W, Philip S. Heterogeneous information network embedding for recommendation. IEEE Trans Knowl Data Eng. 2018;31(2):357–70.

[16] Xu Y, Wang E, Yang Y, Chang Y. A unified collaborative representation learning for neural-network based recommender systems. IEEE Trans Knowl Data Eng 2022;34(11):5126–39. doi: 10.1109/tkde.2021.3054782

[17] Qi L, Hu C, Zhang X, Khosravi MR, Sharma S, Pang S. Privacy-aware data fusion and prediction with spatial-temporal context for smart city industrial environment. IEEE Trans Industr Inform 2020;17(6):4159–67. doi: 10.1109/TII.2020.2971234

[18] Qi L, Wang R, Hu C, Li S, He Q, Xu X. Time-aware distributed service recommendation with privacy-preservation. Inf Sci. 2019;480:354–64.

[19] Fei F, Li S, Dai H, Hu C, Dou W, Ni Q. A k-anonymity based schema for location privacy preservation. IEEE Trans Sustain Comput. 2017;4(2):156–67.

[20] Wu T, Dou W, Wu F, Tang S, Hu C, Chen J. A deployment optimization scheme over multimedia big data for large-scale media streaming application. ACM Trans Multimed Comput Commun Appl (TOMM). 2016;12(5s):1–23.

[21] Long Z, Chen Y, Dou H, Zhang Y, Chen Y. FedSQ: Sparse-quantized federated learning for communication efficiency. IEEE Trans Consum Electr. 2024.

[22] Huang H, Xie L, Liu M, Lin J, Shen H. An embedding model for temporal knowledge graphs with long and irregular intervals. Knowl-Based Syst. 2024;296:111893.

[23] Wang X, Chen H, Zhou Y, Ma J, Zhu W. Disentangled representation learning for recommendation. IEEE Trans Pattern Anal Mach Intell 2023;45(1):408–24. doi: 10.1109/TPAMI.2022.3153112 35196226

[24] Wang X, Jin H, Zhang A, He X, Xu T, Chua T. Disentangled graph collaborative filtering. In: Proceedings of the 43rd international ACM SIGIR conference on research and development in information retrieval. 2020. p. 1001–1010.

[25] Wang X, He X, Cao Y, Liu M, Chua T. KGAT: Knowledge graph attention network for recommendation. In: Proceedings of the 25th ACM SIGKDD international conference on knowledge discovery & data mining. 2019. p. 950–958.

[26] Xin X, He X, Zhang Y, Zhang Y, Jose J. Relational collaborative filtering: Modeling multiple item relations for recommendation. In: Proceedings of the 42nd international ACM SIGIR conference on research and development in information retrieval. 2019. p. 125–134.

[27] Wang X, Ji H, Shi C, Wang B, Ye Y, Cui P, et al. Heterogeneous graph attention network. In: The world wide web conference. 2019. p. 2022–2032. doi: 10.1145/3308558.3313562

[28] Yang L, Liu Z, Dou Y, Ma J, Yu PS. CONSISREC: Enhancing GNN for social recommendation via consistent neighbor aggregation. In: Proceedings of the 44th international ACM SIGIR conference on Research and development in information retrieval. 2021. p. 2141–2145.

[29] Yin L, Wang L, Cai Z, Lu S, Wang R, AlSanad A, et al. DPAL-BERT: A faster and lighter question answering model. CMES 2024;141(1):771–86. doi: 10.32604/cmes.2024.052622

[30] Chen C, Zhang M, Liu Y, Ma S. Social attentional memory network: Modeling aspect-and friend-level differences in recommendation. In: Proceedings of the twelfth ACM international conference on web search and data mining. 2019. p. 177–185.

[31] Yu J, Yin H, Li J, Wang Q, Hung NQV, Zhang X. Self-supervised multi-channel hypergraph convolutional network for social recommendation. In: 2021 Proceedings of the web conference. 2021. p. 413–424.

[32] Shi L, Wu W, Guo W, Hu W, Chen J, Zheng W, et al. SENGR: sentiment-enhanced neural graph recommender. Inf Sci. 2022;589:655–669.

[33] Liu C, Li Y, Lin H, Zhang C. GNNRec: Gated graph neural network for session-based social recommendation model. J Intell Inf Syst. 2023;60(1):137–56.

[34] Tan L, Gong D, Xu J, Li Z, Liu F. Meta-path fusion based neural recommendation in heterogeneous information networks. Neurocomputing. 2023;529:236–48.

[35] Zhang Y, Liao W, Wang Y, Zhu J, Chen R, Zhang Y. Meta-path automatically extracted from heterogeneous information network for recommendation. World Wide Web. 2024;27(3):1–26.

[36] Wang Y, Tang S, Lei Y, Song W, Wang S, Zhang M. DisenHAN: Disentangled heterogeneous graph attention network for recommendation. In: Proceedings of the 29th ACM international conference on information & knowledge management. 2020. p. 1605–1614.

[37] Xia L, Shao Y, Huang C, Xu Y, Xu H, Pei J. Disentangled graph social recommendation. In: 2023 IEEE 39th international conference on data engineering (ICDE). 2023. p. 2332–2344. doi: 10.1109/icde55515.2023.00180

[38] Long X, Huang C, Xu Y, Xu H, Dai P, Xia L. Social recommendation with self-supervised metagraph informax network. In: Proceedings of the 30th ACM international conference on information and knowledge management. 2021. p. 1160–1169.

[39] Tian J, Chang M, Ding Z, Han X, Chen Y. MAHGE: point-of-interest recommendation using meta-path aggregated heterogeneous graph embeddings. In: Proceedings of the international conference on spatial data and intelligence. 2022. p. 250–263.

[40] Ba JL, Kiros JR, Hinton GE. Layer normalization. arXiv Preprint. 2016. doi: arXiv:1607.06450

[41] Chen L, Wu L, Hong R, Zhang K, Wang M. Revisiting graph based collaborative filtering: A linear residual graph convolutional network approach. In: Proceedings of the AAAI conference on artificial intelligence. Vol. 34;2020. p. 27–34.

[42] Wang X, He X, Wang M, Feng F, Chua TS. Neural graph collaborative filtering. In: Proceedings of the 42nd international ACM SIGIR conference on Research and development in Information Retrieval. 2019. p. 165–174.

[43] Wu L, Sun P, Fu Y, Hong R, Wang X, Wang M. A neural influence diffusion model for social recommendation. In: Proceedings of the 42nd international ACM SIGIR conference on research and development in information retrieval. 2019. p. 235–244.

[44] Song W, Xiao Z, Wang Y, Charlin L, Zhang M, Tang J. Session-based social recommendation via dynamic graph attention networks. In: Proceedings of the twelfth ACM international conference on web search and data mining. 2019. p. 555–563.

[45] Chen C, Zhang M, Wang C, Ma W, Li M, Liu Y. An efficient adaptive transfer neural network for social-aware recommendation. In: Proceedings of the 42nd international ACM SIGIR conference on research and development in information retrieval. 2019. p. 225–234.

